# The Antipruritic Effect of Phototherapy

**DOI:** 10.3389/fmed.2018.00333

**Published:** 2018-11-30

**Authors:** Franz J. Legat

**Affiliations:** Department of Dermatology and Venerology, Medical University of Graz, Graz, Austria

**Keywords:** Pruritus, itch, chronic prurigo, prurigo nodularis, phototherapy, UV-light, psoriasis, atopic dermatitis

## Abstract

Phototherapy is widely used to treat inflammatory skin diseases such as psoriasis and atopic dermatitis. Repeated suberythemogenic doses of UV-light reduce inflammation in these diseases and ultimately may lead to a complete disappearance of cutaneous symptoms for weeks or months. Chronic pruritus is an important and highly distressing symptom of many of these inflammatory skin diseases. Interestingly, pruritus is also reduced or completely abolished by UV-treatment of psoriasis and atopic dermatitis, and sometimes reduction of pruritus is the first indication for skin improvement by phototherapy. The cutaneous nervous system is an integral part of skin anatomy, and free nerve endings of sensory cutaneous nerve fibers reach up into the epidermis getting in close contact with epidermal cells and mediators from epidermal cells released into the intercellular space. Stimulation of “pruriceptors” within this group of sensory nerve fibers generates a neuronal signal eventually transmitted via the dorsal root and the spinal cord to the brain, where it is recognized as “itch”. UV-light may directly affect cutaneous sensory nerve fibers or, via the release of mediators from cells within the skin, indirectly modulate their function as well as the transmission of itch to the central nervous system inducing the clinically recognized antipruritic effect of phototherapy.

## Introduction

It has long been recognized that “UV-responsive” skin diseases improve during summer months and worsen during winter, and exposure to natural sunlight, i.e., heliotherapy, is a common way of psoriasis patients to improve their skin lesions. Phototherapy has shown significant effects in these “UV-responsive” skin diseases and is widely used to treat inflammatory skin diseases such as psoriasis, atopic dermatitis (AD) as well as cutaneous T-cell lymphoma (CTCL), e.g., mycosis fungoides/Sezary-Syndrome (1–3). Chronic pruritus (i.e., pruritus lasting for 6 weeks or longer) is an important and highly distressing symptom of many of these inflammatory skin diseases and significantly impairs the quality of life in the affected patients. Repeated suberythemogenic doses of UV-light, as used in phototherapy, are capable of reducing inflammation in these diseases and ultimately may lead to a complete disappearance of cutaneous symptoms for weeks or months. However, not only the skin lesions of these diseases improve but also the accompanying pruritus decreases when patients undergo repeated UV-treatments. Interestingly, phototherapy is capable of improving chronic pruritus in a variety of different pruritic skin diseases beside psoriasis and AD, such as lichen planus, pityriasis lichenoides, urticaria pigmentosa, chronic spontaneous urticaria, parapsoriasis, and CTCL (e.g., Sezary-Syndrome) (4).

Phototherapy, in addition, is also effective against chronic pruritus in systemic diseases such as end-stage renal disease, cholestatic liver disease (e.g., primary biliary cholangitis or cholestatic pruritus of pregnancy), hematologic diseases (e.g., polycythemia vera or Hodgkins lymphoma) and other conditions of chronic pruritus without primary or secondary skin lesions (e.g., drug induced pruritus after hydroxyethyl starch) (4, 5). Even in the various forms of chronic prurigo (6), including the severe nodular and umbilicated ulcer types, as well as in chronic idiopathic pruritus mainly in elderly patients, phototherapy is very effective and sometimes the only treatment improving chronic pruritus (5, 7).

When looking at the broad antipruritic effect of phototherapy the question arises how phototherapy is capable of reducing pruritus in such a variety of inflammatory skin and systemic diseases with obviously very different pathophysiological backgrounds?.

It is clear, that the antipruritic effect of phototherapy has to depend on the ability of UV light to interfere with structures and mediators involved in the induction and perception of pruritus. However, at the moment, the pathophysiology of pruritus in the various skin and systemic diseases is not completely understood and there is even less knowledge about the mechanisms how phototherapy is capable of reducing pruritus in these diseases. In the following paragraphs we try to approach the question of the antipruritic effect of phototherapy by looking at some targets of UV light in the skin and possible UV-induced mediators which may contribute.

## UV-targets in the skin

When UV-light impinges on the skin it reaches the most superficial layers including the cell-rich epidermis as well as the underlying dermis. The longer the wavelength, the deeper UV-light penetrates into the skin. Thus, while the shorter wavelengths of UVB mainly exert their effects in the epidermis and upper papillary dermis, UVA may penetrate into deeper dermal layers. These superficial layers of the skin reached by UV are also the skin layers where pruritus can be perceived (8), and it is a well-known clinical finding, that removal of the superficial skin layers leaves the skin devoid of itch perception, while pain can still be recognized.

In the epidermis, resident cells such as keratinocytes, melanocytes, and Langerhans cells, as well as infiltrating cells such as lymphocytes and leukocytes, can be reached and affected by UV. The connective tissue of the upper dermis, beside fibroblasts and the cells of blood vessels, sweat glands and sebaceous glands, hosts an array of other cells such as lymphocytes, leukocytes, dermal dendritic cells, mast cells, and eosinophils, which are important players in inflammatory and immunological processes.

Within the most upper part of the dermis, just beneath the epidermis, a subepidermal plexus is formed by cutaneous sensory nerves from which nerve fibers perpendicularly grow into the epidermis. As these nerves penetrate the basement membrane they lose their myelin sheath, reach up to the granular layer and stratum corneum and extensively branch within the epidermis. Lying within the intercellular space of the epidermis, these sensory nerves get in close contact with resident keratinocytes, melanocytes and Langerhans cells, or infiltrating lymphocytes and leucocytes. Within this group of intraepidermal sensory nerve fibers (IENF), the pruriceptive sensory nerve fibers, i.e., histamine-sensitive, mechano-insensitive nerve fibers and histamine-insensitive, mechanoheat-sensitive, “polymodal” nerve fibers, can been found. They take up the pruritic signals from the periphery and transmit them via their cell bodies in the dorsal root ganglia (DRG) and their central projections to the spinal cord and further to the brain (8).

UV-light, thus, reaches and may directly or indirectly interact with the dense three-dimensional network of sensory nerves within the epidermis and upper dermis.

**Figure 1:**
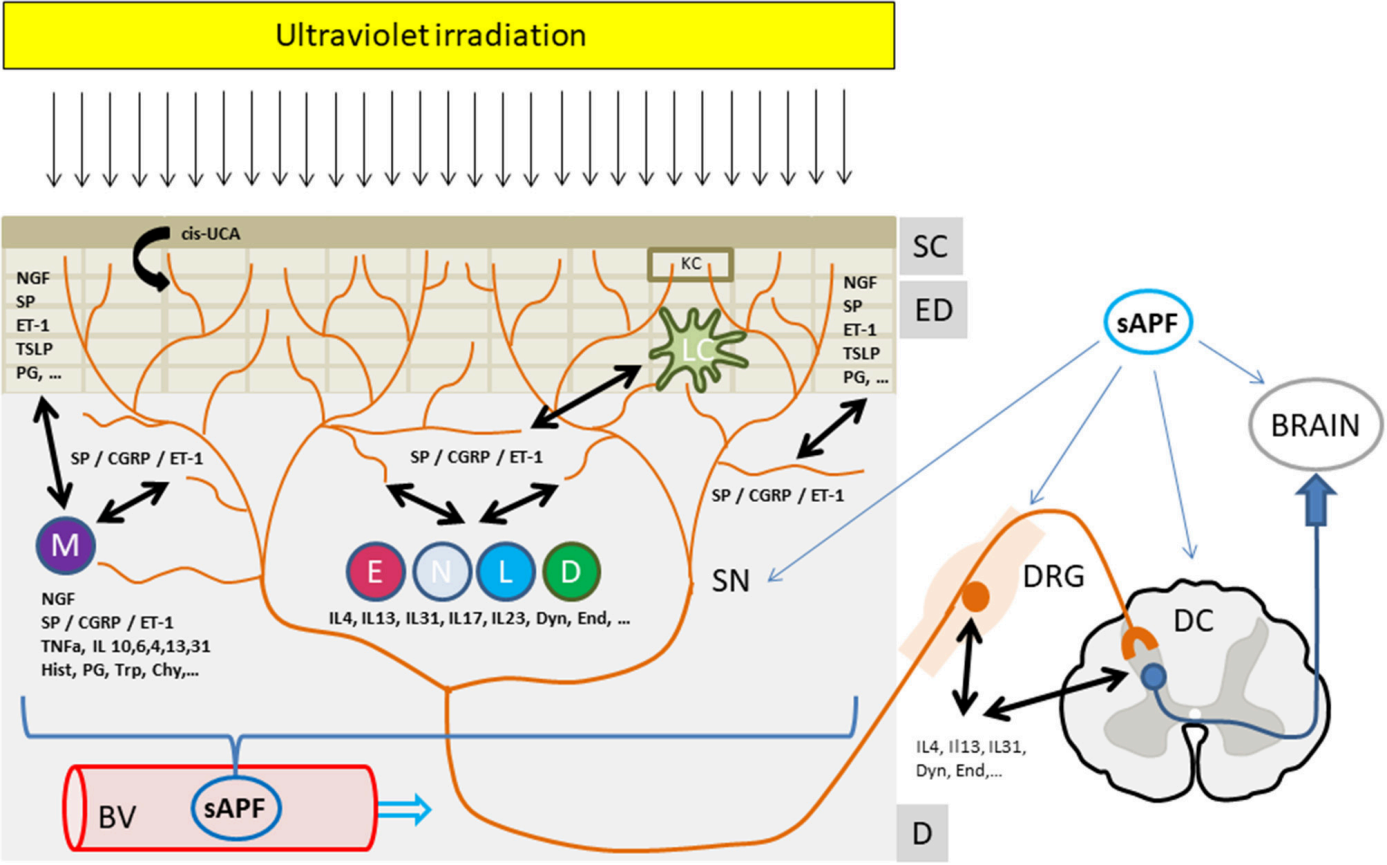
The antipruritic effect of phototherapy. Ultraviolet irradiation reaches and affects all structures and cells within the upper skin layers from the stratum corneum to the epidermal and dermal layers. Upon UV irradiation multiple mediators from sensory nerves, resident or infiltrating cells are affected (decrease, increase, release). These mediators extensively interact with cutaneous nerves and cells eventually leading to an inhibition of itch perception and/or signaling to the brain. In addition, a yet unknown UV-induced “soluble anti-pruritic factor” (sAPF) from the skin may reach the peripheral as well as the central nervous system via the circulation and contribute to the inhibition of itch signaling and/or perception. See text for further details. **Mediators:** Cis-UCA, Cis-urocanic acid; ET-1, Endothelin-1; NGF, Nerve growth factor; CGRP, Calcitonin gene related peptide; SP, Substance P; IL, Interleukin; TNFa, Tumor necrosis factor alpha; Hist, Histamine; PG, Prostaglandins; Trp, Tryptase; Chy, Chymase; TSLP, Thymic stromal lymphopoetin; Dyn, Dynorphin, End, Endorphin; **Structures:** SC, Stratum Corneum; ED, Epidermis; D, Dermis; BV, Blood Vessel; DRG, Dorsal root ganglia; SN, Sensory nerve; DC, Dorsal column, **Cells:** KC, Keratinocyte; M, Mastcell; E, Eosinophil, N, Neutrophil; L, Lymphocyte, D, Dermal Dendritic cell; LC, Langerhans cell.

Both, the interaction with the cellular components as well as with the nerve structures in this skin compartment may convey the antipruritic effects of phototherapy (Figure 1).

## Chronic pruritus and phototherapy

Among the first, who looked into the antipruritic effects of phototherapy in the clinic were Barbara Gilchrest and colleagues. In uremic patients on hemodialysis suffering from chronic pruritus, they could show that repeated broadband (BB)-UVB twice weekly compared to time-matched UVA significantly reduced pruritus in 9 of 10 patients (9). In their studies, they also showed that half-body UVB treatments reduced pruritus not only on the irradiated body half but equally reduced pruritus also on the non-irradiated body-half (10). This indicates that the antipruritic effect of BB-UVB on uremic pruritus in hemodialysis patients is mediated by a systemic, yet unknown effect. In this study they also found that the antipruritic effect is not immediate but requires several treatments and at least 2 weeks to being recognized by the patients. It also occurred that thrice weekly treatments accelerated the onset of the antipruritic effect compared to treatments only once a week, in which the antipruritic effect was not recognized before the 4th week.

In a clinical trial in patients with chronic pruritus with or without pruriginous skin lesions, some of them with renal insufficiency, the antipruritic effect of whole body narrowband (NB)-UVB was not inferior to broadband (BB)-UVB (11). Thus, NB-UVB, today the preferred treatment modality of phototherapy (12), is also effective in treating generalized chronic pruritus.

However, in other skin diseases associated with chronic pruritus such as AD, psoriasis, CTCL or pityriasis rosea, phototherapy with UVB or PUVA exerted a local effect on skin lesions and the associated pruritus (9). In a half-body study in patients with AD, treated with NB-UVB on one half and UVA-1 on the other half, patients were able to recognize differences in pruritus reduction by the two treatments indicating at least a partially local antipruritic effect of NB-UVB and UVA-1. However, an additional systemic effect of the two treatments cannot be excluded and is likely in a half-body study (13). A local antipruritic effect can also be seen in targeted UV-treatments with UVB, UVA-1, or excimer laser (i.e., 308 nm), if single pruriginous nodules or circumscribed lichen simplex chronicus are treated (4).

Thus, it appears that the antipruritic effect of phototherapy involves both local as well as systemic factors, depending on the area of treated skin. This favors the idea of the induction of a soluble antipruritic factor by UVR eventually released into the circulation and affecting peripheral and/or central itch pathways (Figure 1). UV, however, may also locally affect the production and release of itch mediators as well as directly or indirectly change the sensitivity of cutaneous sensory nerves to itch signals. In any case, it has been recognized that only repeated suberythemogenic doses of UV-light induce the antipruritic effect of phototherapy while high doses of UV, especially in the UVB range, induces skin inflammation (“sunburn”) and induces or aggravates pruritus. This implies that the antipruritic effect of phototherapy is also a matter of UV dose and treatment frequency, as shown by Gilchrest et al. (9) in uremic pruritus.

## UV-effects on the opioid system

The group of patients with end-stage renal disease, especially if undergoing hemodialysis, is especially prone to severe pruritus with up to 50% of hemodialysis patients being affected (14). Beside phototherapy with UVB, the systemic application of the μ-opioid receptor antagonists naloxone and naltrexone as well as the kappa-opioid receptor agonist nalfurafine have shown significant antipruritic effects (15). This implies that opioids are important mediators of uremic pruritus and may be among the soluble factors suggested to participate in the “systemic” antipruritic effects of phototherapy in uremic patients.

In addition, topical application of the μ-opioid antagonist naltrexone has shown antipruritic effects in patients with different chronic pruritic disorders (16). Topical application of the kappa-opioid-agonist nalfurafine also showed an antipruritic effect in a murine model of AD (17). Thus, opioids may play a role in both peripheral as well as central modulation of pruritus in uremic pruritus and other pruritic diseases such as AD, in which decrease of kappa-opioid receptors (KOR) but not of μ-opioid receptors (MOR) have been found in the skin, resulting in a misbalance of the MOR over KOR system (18). In AD patients, PUVA has shown to decrease MOR not changing the level of its agonist β-endorphin, but increasing the KOR agonist dynorphin leaving the KOR expression unchanged. Together, these PUVA-induced changes resulted in a decreased activity of the “MOR system” together with an increased activity of the “KOR system,” which correlated with a decreased VAS score for pruritus. The KOR agonist dynorphin is capable of modulating itch perception via e.g., interaction with KOR on interneurons in the spinal cord (19). Thus, an effect of UV on receptors and mediators of the opioid system may contribute to the antipruritic effect of phototherapy in ESRD, AD as well as in other pruritic conditions such as cholestatis, in which the MOR antagonists naloxone and naltrexone have also shown antipruritic efficacy and are recommended in the treatment for cholestasic pruritus (20). Phototherapy has also been reported to be effective in reducing cholestatic pruritus (21), and should be tried in case of resistance to guideline conform treatments.

## UV-induced immunosuppression and the cutaneous nervous system

Systemic immunosuppressive agents such as methotrexate, azathioprine, or mycofenolate mofetil, and especially corticosteroids and cyclosporine, sometimes have shown remarkable antipruritic effects in various diseases such as AD, chronic prurigo, or Sezary-Syndrome, and they are still used in severe recalcitrant cases of chronic pruritus. The mechanisms by which immunosuppressive substances reduce pruritus in these various conditions, however, are not completely understood (22).

Phototherapy with repeated UV irradiations is also capable of inducing local as well as systemic immunosuppression. It is well-known, that the interaction of UV with the cellular components of the skin, mainly by interaction with DNA, leads to a sequence of events resulting in local and systemic immunosuppressive effects such as the suppression of contact hypersensitivity (CHS) and the induction of tolerance, in which T-regulatory cells play an important role (23).

It is less well-known, that the interaction of UV with the cutaneous sensory system also conveys local as well as systemic immunosuppressive effects. The same group of sensory nerve fibers within the epidermis and upper dermis, among which we find the pruriceptive nerve fibers, are also capable of mediating or modulating the immunosuppressive effects of UV.

In mice, acute and chronic UV radiation (UVR) is capable of inducing local and/or systemic immunosuppression (i.e., suppressing CHS). This UV-induced suppression of CHS was blocked in mice with impaired sensory nervous system by pretreatment of these mice with capsaicin on their 2nd day of life (24). Capsaicin is the pungent ingredient of hot chili pepper, which specifically targets capsaicin-sensitive C- and A-delta fibers, leaving rodents insensitive to further capsaicin challenges, if they have been treated with a high dose of capsaicin in the first days of live. In addition, pretreatment with a neuropeptide calcitonin gene-related peptide (CGRP) antagonist, CGRP 8–37, also abolished UV-induced suppression of CHS in mice (25). CGRP is an important neuropeptide within sensory nerve fibers and similarly to UVR is capable of reducing the number of Langerhans cells within the epidermis, which is important in mediating the local immunosuppressive effect of UVR (26). CGRP is often co-localized with substance P (SP), which is an important mediator of neurogenic inflammation via stimulation of neurokinin-1 receptors (NK1R). Both neuropeptides, SP and CGRP, are released by acute high dose UVR resulting in a neurogenic inflammation which contributes to the sunburn reaction (25). However, repeated low doses UVR of mice, increases SP- and CGRP-immunoreactive nerve fibers in the epidermis of irradiated skin compared to non-irradiated skin (27, 28). This increase in neuropeptides within sensory nerve fibers and the increase of the number of intraepidermal nerve fibers are most likely mediated by nerve growth factor (NGF) produced, e.g., by keratinocytes and mast cells upon UVR. NGF, after retrograde neuronal transport from the periphery to the DRG cells, increases the synthesis of neuropeptides and stimulates the outgrowth of sensory nerves in the skin (29). In peripheral inflammation, NGF is increasingly produced and can also induce the release of SP and CGRP from sensory nerve fibers (29). Via a feedback loop, SP acting on NK1R can again increase the production and release of NGF, e.g., by keratinocytes and mast cells. Thus, blocking the NK1R, also a target in antipruritic drug development (e.g., the NK1R antagonist serlopitant in chronic prurigo (30), reduces inflammation as well as NGF production, which may also affect UV-induced immunosuppression. Interestingly, systemic application of NGF is capable of suppressing CHS in mice, and this is abolished in mice with capsaicin-impaired neurosensory systems (31). Anti-NGF antibodies, on the other hand, similarly to the capsaicin-impairment of sensory nerves, are also capable of inhibiting UV-induced suppression of CHS, indicating that NGF and the cutaneous neurosensory system play significant roles in UV-induced immunosuppression.

Another factor mediating systemic immunosuppression by UVR is cis-urocanic acid (UCA), which upon UVB irradiation is converted from the trans-form located within the stratum corneum of the epidermis (32). In mice, cis-UCA similarly to UVR suppresses the induction of CHS (24). Both UVR- and cis-UCA-induced suppression of CHS was reduced in mast cell deficient mice and in mice with capsaicin-impaired neurosensory system. However, cis-UCA is not capable of inducing mast cell degranulation by itself but induces the release of SP and CGRP from cutaneous sensory nerves (24), probably via stimulation of 5-HT2A receptors (33). This may lead to mast cell degranulation and the eventual release of mediators such as TNF-alpha, IL-10 and histamine. Histamine may then stimulate the keratinocyte production of prostanoids, which are important for UV-induced systemic immunosuppression (34).

Thus, it appears that UV-induced immunosuppression is closely related to the cutaneous neurosensory system and a mutual influence of mediators from nerves, keratinocytes, the stratum corneum (e.g., cis-UCA) and mast cells play significant roles in this process. How this is finally translates into antipruritic effects of UVR is not yet known, but the aforementioned mediators involved in UV-induced immunosuppression, play also significant roles in neurogenic inflammation as well as in pruritus.

## Interaction between mast cells and sensory nerves

In the skin, mast cells are located in close proximity to SP and CGRP positive sensory nerves (35). Mast cells are capable of releasing a number of preformed mediators such as histamine and tryptase as well as newly synthesized mediators such as neuropeptides (e.g., SP, CGRP, ET-1, VIP), cytokines (e.g., TNF-a, IL-4, IL-13, and IL-31) and lipid mediators (e.g., leukotriens and prostaglandins). This array of mediators interacts with their respective receptors on neighboring skin cells and sensory nerves, which upon stimulation may release neuropeptides such as SP and CGRP, which act back on mast cells as well as on other cells in the skin. Primary stimulation of sensory nerves and the eventual release of neuropeptides, on the other hand, stimulate the release of mediators from mast cells and other cells in the skin, which again affect cutaneouos sensory nerves. Thus, there is an intensive crosstalk between sensory nerves, mast cells as well as other cells in the skin via the aforementioned and other mediators and their receptors [for review see (35)] and they may participate in the antipruritic effects of UVR (Figure 1).

In lesional skin of AD (36) as well as psoriasis (37) the number of sensory nerve fibers positive for SP and CGRP as well as the number of cutaneous mast cells is increased. In addition, also the contacts between mast cells and SP/CGRP-positive nerves are increased, indicating an intensified crosstalk between nerves and mast cells in AD and psoriasis. Both have a high prevalence of chronic pruritus, especially in lesional skin, and respond well to phototherapy. In the skin of psoriatic patients suffering from pruritus an overexpression of the neuropeptide receptors for SP (NK1R) and CGRP (38) as well as of NGF and its high affinity receptor Trk-A (39) was found. A topical inhibitor of Trk-A, CT327, has shown significant antipruritic effects in psoriatic patients, indicating the importance of NGF for pruritus in psoriasis (40). Similarly, in AD patients an increase in NGF expression and cutaneous nerve fiber density was found. PUVA treatment resulted in downregulation of NGF and decrease of nerve fiber density, as well as in reduction of itch and eczema in these patients (18).

In uremic pruritus patients a papillary dermal “neuropathy” resulting from reduced CGRP+ papillary nerves was observed, which correlated negatively with pruritus intensity, suggesting a preferential loss of pain-sensing CGRP+ papillary nerves. SP+ and natriuretic polypeptide precursor B positive (NNPB+) nerve fibers, however, were preserved and the authors suggested SP+ and NNPB+(CGRP negative)–nerve fibers to be important itch-sensing candidates (41). There was no reduction in intraepidermal nerve fibers in ESRD patients with or without pruritus compared to non-ESRD controls arguing against a small fiber neuropathy causing pruritus in these patients (42).

Wallengren and Sundler reported that in 10 patients undergoing UVB/A, PUVA, or NB-UVB, for different skin diseases a decrease in intra-epidermal PGP9.5–positive nerves and dermal CGRP-positive nerves was shown, but nerve fibers for the vanilloid-receptor 1 (VR1) were not affected (43). They postulated that the reduction in nerve fibers by phototherapy may be responsible for the reduction of itch detected in these patients.

This is in discrepancy to the aforementioned increase in SP/CGRP-positive cutaneous nerve fibers by repeated suberythemogenic UVB irradiation in mice (27, 28) as well as to the hypothesis of Du et al. (41), that a reduction of CGRP+ nerves in the papillary dermis may participate in uremic pruritus. An increase in intraepidermal nerve fibers, SP and CGRP, as well as NGF, but a reduction of NK1R was also found in chronically sun-exposed skin by Toyoda et al. (44). Thus, there are conflicting results about a decrease or an increase in the number of cutaneous nerve fibers after repeated (suberythemogenic) UVR or phototherapy in mice and humans.

An increased number of mast cells was also found in the skin of patients with uremic pruritus. *In-vitro* experiments, showed an increased apoptosis of mast cells by BB-UVB and NB-UVB, suggesting a role of UV-induced MC-apoptosis in the antipruritic effect of phototherapy, at least in uremic pruritus (45). Indeed, a decrease in the number of mast cell as well as in pruritus after 2 months of UVB treatment was found in patients with uremic pruritus by Cohen et al. (46), however, the authors did not find a clear correlation between the reduction of mast cells and pruritus.

In urticaria pigmentosa, with a significant increase in mast cells in the skin of patients often accompanied with intense pruritus, PUVA is capable of reducing the number of cutaneous mast cells (47) as well as pruritus. In a study treating urticaria pigmentosa patients with high- and medium-dose of UVA-1, mast cells as well as pruritus also significantly decreased (48).

Taken together, it is not yet clear whether the change in the number of cutaneous nerves and/or mast cells is directly related to an antipruritic effect of phototherapy. It, however, shows, that UVR as applied by phototherapy is capable of affecting these two important players and thus affects pruritus, e.g., by mediators derived from them.

Endothelin-1 (ET-1) is such a mediator and neuropeptide. It is released from sensory nerves and by a number of skin cells including vascular endothelial cells, keratinocytes and mast cells, and is capable of inducing itch (49). In addition, stimulation of mast cells by ET-1, similar to SP, induces the release of several mediators such as histamine, leukotriens, IL-6, and TNF-a. On the other hand, ET-1 also stimulates the release of mast cell chymase, which degrades ET-1 and thus protects against ET-1 abundance, a condition which in mast cell deficient mice resulted in hypothermia, diarrhea and an increased death rate after systemic application of ET-1 (50).

Via this pathway, mast cells may even play an antagonistic effect against itch induced by UVR. Schweintzger et al. (51) have shown that, compared to normal mice, mast cells deficient KitW-Sh/W-Sh mice developed a specific photo-induced pruritus shortly after UV irradiation with doses well below inflammatory “sunburn” doses. Reconstitution of these mice with mast cells abolished this phenomenon of “photo-itch.” The authors explained this mast cell dependent UV-induced pruritus with an accumulation of ET-1 in the skin, induced by UVR (52), that resulted from an insufficient inactivation of ET-1 by the absence of mast cells-derived ET-1-degrading enzymes. The unopposed increase of ET-1 eventually may have stimulated cutaneous sensory nerves via their specific ETA receptors (49) causing the described photo-itch.

Other mast cells derived mediators may also stimulate pruritus. Beside mediators such as histamine, TNF-a, and IL-10, the enzyme tryptase is released upon mast cell stimulation and is capable of activating specific “protease activated receptors” (PAR2) on sensory nerve fibers or keratinocytes. By cleaving a tethered ligand of PAR, auto-activation of the receptor eventually causes the release of neuropeptides such as SP and CGRP, inducing neurogenic inflammation as well as pruritus (53). In AD, as aforementioned, the number of mast cells, SP- and CGRP-positive sensory nerves as well as NGF is increased (18, 36), and tryptase is upregulated. The release of tryptase from mast cells by NGF, eventually activating PAR2 on sensory nerves, thus, may also play a role in pruritus of AD (35).

## Role of cytokines in the antipruritic effect of phototherapy

Cytokines released from various cutaneous cells such as keratinocytes, Langerhans cells, mast cells, eosinophils and infiltrating lymphocytes are also suggested to be important mediators in chronic pruritus. Among these cytokines some are of specific interest.

In psoriasis, e.g., TNF-a, IL-17, and IL-23, are increased in the skin and may play a role in chronic pruritus of psoriatic patients. More than 80% of all patients suffer from chronic pruritus, and pruritus is the most distressing symptom of this disease (54). In clinical trials investigating anti-psoriatic treatments such as “biologicals” targeting these cytokines or their receptors (e.g., TNF-alpha or its receptor, IL-12/23p40, IL-23p19, and IL-17 or its receptors), beside an anti-psoriatic effect also a significant antipruritic effect of these drugs was detected. In addition, the “small molecules” such as phosphodiesterase 4 (PDE4) or Janus kinase (JAK) inhibitors have shown significant antipsoriatic as well as antipruritic effects. The reduction of pruritus by these biologicals or small molecules often paralleled or even preceded the reduction of psoriatic skin lesions (55).

Though the exact pathophysiology of pruritus in psoriasis is not yet known, it can be assumed that TNF-a, IL-17, and IL-23, may be involved. Indeed, e.g., the main receptor for IL-17A is found on many neural tissues and IL-17A participate in several neuroimmune interactions and directly or indirectly interact with neuronal functioning on the level of the DRG and the spinal cord. In addition, TNF-alpha may enhance the excitability of DRG neurons to other stimuli (56). In means of phototherapy, NB-UVB, the most frequently used phototherapy for psoriasis, has shown a significant downregulation of IL-17 in lesional as well as perilesional skin of vitiligo patients (57). In addition, PUVA therapy in psoriasis patients resulted in a significant downregulation of IL23 (IL12/23p40 and IL23p19). This indicates that phototherapy is capable of downregulating IL-17 as well as IL-23, and similarly to blockade of IL-17 or IL-23 with biologicals, this may contribute to the antipruritic effects of phototherapy, at least in psoriasis.

Another interesting cytokine is IL-31, which is primarily secreted by T-cells, mast cells, eosinophils, dendritic cells, and macrophages. Mast cell as well as eosinophil degranulation, e.g., by SP, may increase on-site IL-31 concentrations. IL-31, then binding to its receptor on sensory nerves can induce itch, and may also promote growth of nerves. It has been shown, that IL-31 induced pruritus is mediated via Transient Receptor Potential (TRP) receptors TRPV-1 and TRPA-1 (58).

In recent clinical trials, the IL-31Ra antagonist nemolizumab was capable of significantly reducing pruritus in AD (59) and in addition, improved atopic eczema. However, it is believed that IL-31 is also involved in pruritic conditions of other origin such as chronic prurigo, psoriasis, and cutaneous T-cell lymphoma (60). All of these conditions significantly respond to phototherapy and, thus, the question arises whether phototherapy also affects IL-31 or IL-31Ra. While acute high dose UVB is capable of transiently increasing IL-31 expression in the skin (61), UVA-1 phototherapy with suberythemogenic therapeutic doses for 6 weeks reduced IL-31 mRNA expression to levels close to normal, beside reducing atopic eczema and pruritus (62). In psoriasis, it has been shown that 20 repeated suberythemogenic NB-UVB treatments significantly reduced IL-31 serum levels (63). Thus, while acute high dose UVB increased IL-31 and pruritus, repeated lower doses of UVA-1 and NB-UVB appear to reduce IL-31 and pruritus, and it may be speculated that IL-31 reduction in the skin may contribute to the antipruritic effect of phototherapy in AD, in psoriasis, and maybe other pruritic conditions, e.g., chronic prurigo and CTCL, in which increased IL-31 or its receptor appear to play a role in chronic pruritus.

Other important interleukins, especially in AD, are IL-4 and IL-13, and it has been shown, that beside the aforementioned expression of IL-31, also IL-13 expression was reduced by UVA-1 phototherapy in AD patients (62). As aforementioned, the importance of IL-4 and IL-13 in AD was highlighted by the newly developed and already licensed antibody dupilumab, which targets the IL-4-receptor alpha-chain of the heterodimeric IL-4 and IL-13 receptors, and, thus, blocks both IL-4 and IL-13 mediated effects, which has shown significant antipruritic and anti-eczematous effects in AD patients (64). While both, IL-4 and IL-13, has been shown to directly stimulate a subset of DRG neurons *in vitro*, intra-cutaneous injection of IL-4 or IL-13 did not induce acute pruritic responses in mice (7). However, IL-4 enhanced neural responsiveness to multiple pruritogens such as histamine, chloroquine, thymic stromal lymphopoetin (TSLP) or IL-31. This increase in responsiveness to pruritogens was mediated via neuronal Janus kinase (JAK)-1. The authors reported that inhibition of JAK-1 by ruxolitinib or deletion of neuronal JAK-signaling in mice significantly reduced scratching in a murine AD model even in the presence of skin inflammation. In humans, tofacitinib, a JAK-1/3 inhibitor, significantly reduced pruritus in chronic idiopathic pruritus patients (7), who also favorably respond to phototherapy. The authors concluded that IL-4, via neuronal JAK-1, is an important mediator of chronic pruritus as it “sensitizes” pruriceptive sensory nerves and lowers the threshold for other prurigenic mediators to induce itch. Interestingly, these authors also showed that like the activation of sensory nerves by IL-31, the TH2 cytokines IL-4 and IL-13 directly activate pruritic sensory nerves via TRP-channel dependent calcium influx.

Thus, the TRPV1 receptor, which is the classical capsaicin-receptors, appears to play a central role in mediating the effects of the important cytokines IL-31, IL-4, and Il-13, which seems to be crucial in chronic pruritus and eczema formation in AD, one of the major diseases treated successfully with phototherapy. In fact, it has been shown, that inhibition of TRPV1 receptors is capable of blocking pro-inflammatory effects of acute high dose UVR such as the induction of mRNA expression of the pro-inflammatory cytokines IL-1ß, IL-2, IL-4, and TNF-a as well as COX-2, indicating that UVR is indeed capable of affecting TRPV1 receptors (65). However, the effect of repeated suberythemogenic UVR, as used in phototherapy, on TRPV1 receptors is not yet known.

## Conclusion

In conclusion, phototherapy has been shown to have significant antipruritic effects in various pruritic skin diseases in clinical trials and daily practice. Phototherapy also reduces pruritus in systemic diseases without primary skin lesions. Critical for the local or systemic antipruritic effect of phototherapy is the total area of skin irradiated, the number of UV treatments as well as the UV-dose. While high doses of UV result in sunburn and induction or aggravation of pruritus, repeated suberythemogenic UV doses are capable of inducing an antipruritic effect.

Despite the fact, that in recent years more and more information on possible mediators and receptors of chronic pruritus in various skin and systemic diseases became available, the exact pathophysiology of chronic pruritus in these diseases is not completely known, and at the moment our understanding about the possible mechanisms by which phototherapy conveys its antipruritic effect is very fragmented. Future laboratory and clinical investigations addressing the specific question, how repeated UV irradiation may affect cellular and neuronal structures and mediators involved in chronic pruritus, are necessary to combine the pieces of the puzzle to a clearer “image” of the antipruritic effect of phototherapy.

## Author contributions

The author confirms being the sole contributor of this work and has approved it for publication.

### Conflict of interest statement

The author declares having participated in clinical trials investigating antipruritic medications and received travel grants and/or honoraria for oral presentations as well as for participations in Advisory port meetings from Abbvie, Celgene, Janssen, Galderma, Leo Pharma, Menlo, Trevi, and Pfizer.
